# Supplementary material for: From sequence to enzyme mechanism using multi-label machine learning
Source: BMC Bioinformatics. 2014 May 19;15:150. doi: 10.1186/1471-2105-15-150 (PMC4229970; doi:10.1186/1471-2105-15-150)
Supplement: Additional file 2 — Java code of ml2db. Additional file ml2db_code.tar.gz contains the Java source code to run the multi-label machine learning experiments and save the results to database. The code’s Javadoc is included. [file 1471-2105-15-150-S2.zip › additional file 2/ml2db/ml2db/doc/test/dataharness/package-summary.html]

test.dataharness


---


|  |  |  |
| --- | --- | --- |
| **Overview** \| **Package** \| Class \| **Use** \| **Tree** \| **Deprecated** \| **Index** \| **Help** | |  |
| **PREV PACKAGE**   **NEXT PACKAGE** | **FRAMES**    **NO FRAMES**     **All Classes** |  |


---

## Package test.dataharness

| **Class Summary** | |
| --- | --- |
| **ArffPropsFilesTest** | Test the ARFF properties files used in the test harness and their content. |
| **ArffPropsOneTest** | Get the arff data properties from file |
| **ArffPropsQueriesOneTest** | Class |
| **ArffPropsQueriesTest** | Checks the results of the queries contained in the arff properties files |
| **ArffPropsQueriesTwoTest** | Class |
| **ArffPropsTwoTest** | Class |
| **CreateDataTable** | Create a table for test instances (and their attributes and classes) |
| **DatabaseTest** | Check the test database |
| **DataOne** | Class |
| **DataTableOneTest** | Basic data table containing 3 columns with: instances, attributes, classes. |
| **DataTableThreeTest** |  |
| **DataTableTwoTest** |  |
| **DataTwo** | Class |
| **LoadFirstValidArff** | Class |
| **TestProjectParameters** |  |
| **TestProjectParametersTest** | Class |

---


|  |  |  |
| --- | --- | --- |
| **Overview** \| **Package** \| Class \| **Use** \| **Tree** \| **Deprecated** \| **Index** \| **Help** | |  |
| **PREV PACKAGE**   **NEXT PACKAGE** | **FRAMES**    **NO FRAMES**     **All Classes** |  |


---
